# Evaluation of Rumen Degradation Kinetics of Low-Lignin Alfalfa ‘Hi-Gest^®^ 360’ in Saskatchewan Canada

**DOI:** 10.3390/ani13061047

**Published:** 2023-03-14

**Authors:** Daalkhaijav Damiran, Bill Biligetu, Herbert Lardner

**Affiliations:** 1 Department of Animal and Poultry Sciences, University of Saskatchewan, Saskatoon, SK S7N 5A8, Canada; 2 Department of Plant Sciences, College of Agriculture and Bioresources, University of Saskatchewan, Saskatoon, SK S7N 5A8, Canada

**Keywords:** low-lignin alfalfa, maturity, mixtures, degradation kinetics

## Abstract

**Simple Summary:**

Digestibility (or degradability) of alfalfa by animals is hampered by its indigestible type of fiber called lignin. Recently, Alforex Seeds (Woodland, CA, USA) released a low-lignin cultivar called Hi-Gest^®^ 360. We studied degradability of ‘Hi-Gest^®^360’ in monoculture and binary mixtures. Two cultivars of alfalfa (Hi-Gest^®^360 and AC Grazeland) and their binary mixtures with hybrid bromegrass (cv. AC Success) were cut at three maturity stages of alfalfa (10%, 40%, and 100% bloom) and were incubated in cannulated cow’s rumen. Protein and fiber disappearances were calculated as the difference between original and residue amounts of alfalfa after ruminal incubation. Decline in degradability as maturity stage advanced in the forages was evident. The ‘Hi-Gest^®^360’ had 8.6% less lignin compared to AC Grazeland. The HiGest possessed an average of 13% greater fiber and 5% greater dry matter degradability relative to AC Grazeland. Moreover, ‘Hi-Gest^®^360’ maintained quality for a longer time. This finding indicated that delaying harvest of ‘Hi-Gest^®^360’ alfalfa by up to two weeks did not affect the digestibility of nutrients. Thus, the results suggest that if digestibility (or quality) of forage and wider harvest window and/or later harvest is the main concern for producers, then ‘Hi-Gest^®^360’ may be a better alternative among alfalfa cultivars.

**Abstract:**

The objective of this study was to determine rumen degradation kinetics of new low-lignin alfalfa (*Medicago sativa* L.) cv. Hi-Gest^®^360 (HiGest) in comparison with conventional alfalfa cv. AC Grazeland (Grazeland) in monoculture and binary mixtures at different maturity stages. Two cultivars of alfalfa (HiGest, and AC Grazeland) and their binary mixtures with hybrid bromegrass (HBG; cv. AC Success), grown in 2019 at two locations (Saskatoon and Lanigan), were cut at three maturity stages of alfalfa (1 = 10% bloom; 2 = 40% bloom; and 3 = 100% bloom). Rumen degradation characteristics, including rapidly degradable fraction (S), potentially degradable fraction (D), undegradable fraction (U), degradation rate (K_d_), lag time (T_0_), and effective degradability (ED) of each component were determined using in situ technique and were analyzed by a first-order kinetic equation described by Ørskov and McDonald with lag time. Generally, in alfalfa monoculture, S or D were decreased and U was increased without affecting K_d_ and T_0_, resulting in decreased ED fraction with increasing stage of maturity. In binary mixtures, plant maturity stages have negligible effects on rumen degradation characteristics of CP. HiGest had higher effective degradability of DM (EDDM) as well as of NDF (EDNDF) than Grazeland. In conclusion, HiGest had greater DM and NDF rumen degradation potential relative to Grazeland. HiGest and Grazeland were different in DM and CP degradation patterns, with HiGest having higher EDDM and EDCP than Grazeland.

## 1. Introduction

Alfalfa (*Medicago sativa* L.) is widely used as forage for livestock due to its high nutrient content [1,2]. However, digestibility and utilization of alfalfa by animals are hampered by its lignin content [3,4]. Lignin is a complex structural polymer that is found in the secondary cell walls of many plants. It is the second most abundant organic compound on Earth, after cellulose, and is an important component of plant biomass. Lignin is responsible for providing rigidity and strength to plant cell walls, which is essential for plants to stand upright and resist external stresses [5]. As a plant matures, the concentration of lignin in the secondary cell walls of its tissues increases. This process is known as lignification and is a natural part of the process of plant growth and development. Lignin is deposited in the spaces between cellulose, hemicellulose, and pectin molecules in the secondary cell wall, where it forms a network of cross-linkages with the other components of the cell wall. This cross-linking helps to increase the strength and rigidity of the cell wall, allowing the plant to stand upright and resist external stresses [6,7]. While lignin is essential for normal plant growth and development, excessive lignification in forage crops such as alfalfa can reduce their feeding value for livestock by decreasing the digestibility of the plant material. Lignin is a complex structural polymer that is resistant to microbial degradation, and its deposition in plant cell walls can make the cellulose and hemicellulose components of the cell wall less accessible to rumen microbial enzymes, which can reduce the efficiency of rumen fermentation and decrease the availability of nutrients to the animal. In addition, lignin can inhibit the activity of digestive enzymes in the small intestine, which can further reduce the digestibility of the feed [8]. Plant breeders are working to develop new varieties of forage crops that have reduced lignin content while maintaining other desirable traits, such as yield and disease resistance [9]. Recently, Alforex Seeds (Woodland, CA, USA) released a low-lignin alfalfa cultivar Hi-Gest^®^ 360, which is a product of conventional plant breeding. Low-lignin alfalfa may provide high-quality forage for livestock in selected seasons or backgrounding programs, allowing producers to graze earlier than other conventional cultivars with improved animal performance. Rumen degradability is a critical factor in the nutritional evaluation of feedstuffs for ruminant animals [2,10,11]. The objective of the study was to investigate the rumen degradation kinetics of the new alfalfa cultivar ’Hi-Gest^®^360’ in monoculture and binary mixtures under the climate conditions of western Canada.

## 2. Materials and Methods

### 2.1. Forage Sample Collection

The forage samples were derived from a field plot study, which evaluated low-lignin alfalfa (*Medicago sativa*) cv. Hi-Gest^®^360 (HiGest) for forage yield, nutrient profile, and establishment costs, available in Damiran et al. [9]. Briefly, the field trials were established in 2017 at two sites: (i) Agriculture and Agri-Food Canada Saskatoon Research and Development Centre, Saskatoon (lat 52°07′ N, long 106°38′ W) with Orthic Dark Brown Chernozem soil and (ii) Termuende Research Ranch (lat 51°51′ N, long 105°02′ W) of Livestock and Forage Centre of Excellence, Lanigan, Saskatchewan, on section 21 Field 8 with Chernozemic Black Oxbow soils [9]. Forty-eight plots (individual plots were 1.2 × 6 m in size) in each site were randomly assigned to 1 of 4 replicated (*n* = 4) treatments: two cultivars of alfalfa (*Medicago sativa* L. cv. AC Grazeland (Grazeland) and Hi-Gest^®^ 360 (HiGest)) in monoculture or in binary mixtures with AC Success hybrid bromegrass (HBG) (Grazeland-HBG and HiGest-HBG) with three maturity stages (stage) of alfalfa (1 = 10% bloom; 2 = 40% bloom; and 3 = 100% bloom). It is common practice in the western Canadian forage industry to categorize alfalfa growth stages into three stages: stage 1, stage 2, and stage 3. Stage 1 is typically defined as the period approximately one week before the point where the hay would be commercially harvested. Stage 2 is at the point of commercial cutting, where the alfalfa reaches the optimal stage of growth for hay production. Stage 3 is approximately one week after the commercial cutting stage [9]. In 2019, at the Saskatoon site, the cutting date was 8, 12, and 19 July for the maturity stages 1, 2, and 3 of alfalfa, respectively. The plots were harvested using a WinterSteiger forage harvester (WinterSteiger, Salt Lake, UT, USA). At the Lanigan site, cutting date was 27 June, 8 July, and 29 July for the maturity stages 1, 2, and 3 of alfalfa, respectively. At Lanigan, forages were cut using a Jari Mower [9]. At each sampling time, sub-samples of approximately 2 kg were taken from each plot and placed in paper bags. These sub-samples were then weighed while still fresh and were subsequently dried in a forced-air oven at a temperature of 55 °C and were evaluated for rumen degradation kinetics.

### 2.2. Experimental Animals

Eight Black Angus cows (876 ± 34 kg) (mean ± STD) fitted with rumen cannulae (13 cm i.d.; Bar Diamond Inc., Parma, ID, USA) were housed in outdoor drylot pens (50 × 120 m) located at the Termuende Research Ranch, Livestock and Forage Centre of Excellence, University of Saskatchewan. The cannulated cows were fed grass-alfalfa hay (TDN 61.0 ± 3.8%, CP 10.6 ± 2.4%, NDF 59.8 ± 5.9%). The cows were adapted to the diet for 21 d prior to the in situ rumen incubation study. All animals were supplied water by heated water bowl. All cows were fed and had ad libitum access to a commercial 2:1 mineral supplement (Right Now^®^ Bronze, Cargill Nutrition, Winnipeg, MB, Canada) and a cobalt iodised salt block (The Canadian Salt Company Ltd., Pointe-Claire, QC, Canada). The trial was pre-approved by the Animal Care Committee of the University of Saskatchewan (protocol number 20100021), and all animals were managed according to the Canadian Council of Animal Care Guidelines [12].

### 2.3. In Situ Rumen Incubation

The study was conducted according to the in situ procedure as described in Ørskov and MacDonald [13] and in Damiran and Yu [14]. All forage samples were ground to pass through a 2 mm screen using a Thomas–Wiley Laboratory Mill (Model 4, Thomas Scientific, Swedesboro, NJ, USA). Seven grams of sample was weighed into number-coded, commercially made nylon bags (5 × 10 cm, #BG510, Bar Diamond Inc., USA) with 40 µm pores, and bags were then sealed. The weights of the bag and forage sample were recorded for each respective sample. The nylon bags with sample were randomly allocated to the eight cannulated cows and placed inside 8 mesh laundry bags before they were inserted into the rumen. Each bag was secured with an 80 cm cord that extended outside of the cannulae plug. Samples were incubated for 0, 4, 8, 12, 24, 48, and 72 h according to the gradual addition/all out procedure described in Damiran and Yu [14]. At the end of incubation, all bags were removed from the rumen at the same time, and excess ruminal contents were removed by a stream of cold tap water. Following this, all samples including 0 h incubation bags were rinsed in cold tap water in six plastic tubs, and excess water was removed by gently pressing the rinsed samples. The rinsed bags (with sample residue) were then dried in a forced air oven at 55 °C for 48 h. All residue samples were weighed after drying, removed from the bags, and pooled according to replicate (plots) of each forage, sampling date, and incubation duration. All pooled residues were ground to pass through a 1 mm screen with a Wiley mill grinder (Model #2, Arthur H. Thomas Co., Philadelphia, PA, USA). Dry matter, CP, and NDF disappearance were calculated as the difference between original and residue amounts after ruminal incubation.

### 2.4. Analysis

Forage samples and residues from in situ incubation were analyzed for DM, CP, and NDF. Dry matter (DM; AOAC method # 930.15), and crude protein (CP; AOAC method # 984.13) contents were analyzed according to the procedure of AOAC [15]. Crude protein was determined using a Leco FP-2000 nitrogen analyzer (Leco Corporation, St. Joseph, MI, USA), neutral detergent fiber with heat stable α-amylase (NDF) was analyzed according to the procedures of Van Soest et al. [16] using an ANKOM Fiber Analyzer (ANKOM Technology Corporation., Fairport, NY, USA).

### 2.5. Rumen Degradation Kinetics

The rumen degradation characteristics included rapidly degradable (soluble) fraction (S, g kg^−1^), potentially degradable fraction (D, g kg^−1^), which was degraded exponentially, undegradable fraction (U, g kg^−1^), the rate of degradation (K_d_, h^−1^) and lag time (T_0_, h), effective degradability (EDDM, g kg^−1^ DM), and rumen undegradable DM (RUDM, g kg^−1^ DM).

In situ data were fitted to the first-order kinetic degradation model [13]:

R (t) = U + D × exp (− K_d_ × (t − T_0_)) (1)

where R (t) is the amount of residue at t hour of incubation (g kg^−1^), U is the undegradable fraction (g kg^−1^), D is the potentially degradable fraction (g kg^−1^), T_0_ is the lag time (h), and K_d_ is the degradation rate (h^−1^). This model described the rumen degradation of DM, CP, NDF and was solved with the use of the NLIN procedure of SAS with iterative least-square regression [14]. Effective degradability (ED) of each component (DM, CP, and NDF) was determined using the nonlinear (NLIN) parameters and was calculated by the equation (S, U, D, and K_d_) [13] as:

ED (g kg^−1^) = S + D × K_d_/(K_p_ + K_d_) (2)

where S is the soluble fraction (g kg^−1^), and a passage rate (K_p_) value of 0.05 h^−1^ was used to represent the rumen turnover rate. In NDF, rumen degradation was described by assuming two fractions (D and U) with lag time T_0_ [15].

### 2.6. Statistical Analysis

Statistical analysis was performed using the GLIMMIX procedure in SAS version 9.4 [14]. Differences among environments, and mainly agrotechnical differences of stand establishment, resulted in significant interactions between locations and cutting stages; therefore, data were analyzed by locations and were reported separately. The replicate was considered as a random effect; cutting treatment and cultivar were designated as fixed effects. Therefore, the model used for the analysis was:

*Y*_jk(i)_ = µ + *F*_i_ + *V*_j(a)_ + *V*_j(t)_ + *M*_k(a)_ + *M*_k(t)_ + e_ijk_

where *Y*_jk(i)_ is an observation of the dependent variable for the forage (entry)*j* at maturity stage *k* in the forage *i*; µ is the population mean for the variable; *F*_i_ is the forage type *i*, *i* = *a*, *t*; a is for monoculture, and *t* is for binary mixtures with HBG; *V*_j(a)_ is the effect of an alfalfa cultivar (Grazeland and HiGest) nested within monoculture; *V*_j(t)_ is the effect of forage mixture (Grazeland-HBG and HiGest-HBG) nested within binary mixtures; *M*_k(a)_ is the effect of forage maturity nested within monoculture; *M*_k(t)_ is the effect of forage mixture nested within binary mixtures; and e_ijk_ is the random error associated with the observation *jk(i)*. Treatment contrasts [14] (monoculture vs. binary; Grazeland alfalfa vs. HiGest alfalfa; Grazeland-HBG vs. HiGest-HBG; Stage 1 vs. Stage 2; Stage 1 vs. Stage 3; Stage 2 vs. Stage 3) were used to determine treatment differences. The plots were replications included as random effects for statistical analysis. Individual samples from each plots comprised the experimental unit, and statistical significance was set at *p* ≤ 0.05. No attempt was made to compare results across sites because they are not central to the objective of evaluating the cultivars included in this study. Therefore, data are presented by forage and cutting stage within the site. If data were unbalanced, pooled standard error was calculated and reported.

## 3. Results

### 3.1. Chemical Composition

Forage CP and NDF concentrations at the Saskatoon and Lanigan sites are presented in Table 1. No significant interactions (*p* > 0.05) between cutting treatments, cultivars, and mixtures were found for nutritive value and rumen degradation kinetics. Therefore, the main effects of cutting treatment and cultivar × mixtures are reported (Table 1).

HiGest and Grazeland alfalfa did not differ (*p* > 0.05) in either NDF or CP in both sites. There were no differences observed (*p* > 0.05) in either NDF or CP between binary mixtures. Alfalfa monoculture had greater (*p* < 0.01) CP but had lower NDF relative to binary mixtures in both sites. In binary mixtures, as alfalfa growth stage advanced (stages 1 to 3), CP decreased (*p* < 0.05) and NDF increased (*p* < 0.05) at the Lanigan site. No difference (*p* > 0.05) was observed in CP among maturity stages in binary mixtures. At the Saskatoon site, binary mixtures at stage 1 had greater NDF compared to both stage 2 and stage 3. Overall, both CP and NDF differences among binary mixtures or among monocultures were minimal or inconsistent at the sites.

### 3.2. The Rumen Degradation Kinetics of DM

The DM rumen degradation kinetics of forages in Saskatoon site is presented in Table 2. HiGest had a lower (11.8% less; *p* < 0.001) undegradable DM fraction (246 vs. 279 g kg^−1^ DM) and hence had a greater (*p* = 0.014) degradable DM fraction (501 vs. 452 g kg^−1^ DM), as well as EDDM (621 vs. 603 g kg^−1^ DM) than AC Grazeland. The stage of maturity at harvesting had a large affect on forage degradability. Generally, in alfalfa monoculture, as the stage of maturity advanced, the soluble fraction S decreased, and the undegradable fraction U of DM increased. The EDDM and therefore RUDM in stage 2 and stage 3 were different (*p* = 0.04) from each other. In binary mixtures, forages harvested in three stages differed for the S fractions with the value being greatest in stage 2 (211 g kg^−1^ DM), intermediate in stage 3 (190 g kg^−1^ DM), and the lowest in stage 1 (171 g kg^−1^). Lag time as well as K_d_ were not affected (*p* > 0.05) by maturity stage. Alfalfa had higher (*p* < 0.05) K_d_ (0.15 vs. 0.06 h^−1^), soluble fraction S (261 vs. 191 g kg^−1^ DM), potentially degradable fraction D (477 vs. 414 g kg^−1^ DM) and had lower undegradable fraction U (263 vs. 395 g kg^−1^ DM) and lag time (0.17 vs. 0.53), and consequently greater effective degradability of DM (EDDM) fraction (612 vs. 408 g kg^−1^ DM) relative to alfalfa-HBG.

At the Lanigan site, HiGest had lower (*p* < 0.05) U (14.7% less; 320 ± 8.4 vs. 375 ± 8.9 g kg^−1^ DM) and RUDM (7.0% less; 479 ± 5.8 vs. 515 ± 8.9 g kg^−1^ DM) and hence had a greater EDDM (7.2% more; 520 ± 5.8 vs. 485 ± 8.9 g kg^−1^ DM) and T_0_ (0.88 ± 0.23 vs. 0.26 ± 0.14 h^−1^) relative to AC Grazeland (Table 3). Generally, in alfalfa monoculture, as the stage of maturity advanced, the degradable fraction D decreased, and the undegradable fraction U and T_0_ increased; consequently, EDDM was decreased from 520 to 493 g kg^−1^ DM for stage 1 and stage 3, respectively. Both in monoculture and binary mixtures, HiGest at stage 3 was similar (*p* > 0.05) with Grazeland at stage 2 in EDDM and therefore in RUDM. In monoculture, HiGest at stage 3 had similar EDDM (516 vs. 511 g kg^−1^ DM) relative to Grazeland at stage 1 (data not shown). In binary mixtures, as the stage of maturity progressed, for T_0_ (from 1.44 to 0.07 h), the degradable fraction decreased (from 491 to 333 g kg^−1^ DM; decreased by 47.4%), the undegradable fraction U (from 334 to 483 g kg^−1^ DM; by 44.6%) increased, and the EDDM decreased from 408 to 369 g kg^−1^ (by 10.6%) DM for stage 1 and stage 3, respectively. The lag time, K_d_, D, U, EDDM, as well as RUDM fractions were not affected by treatment in binary mixtures (AC Grazeland-HBG vs. HiGest-HBG) and averaged 0.83 ± 0.18 h, 0.05 ± 0.004 h^−1^, 438 ± 16.8 g kg^−1^ DM, 367 ± 19.5 g kg^−1^ DM, 388 ± 4.5 g kg^−1^ DM, and 611 ± 4.5 g kg^−1^ DM (mean ± SD), respectively. Alfalfa had higher (*p* < 0.05) K_d_ (106.5% more; 0.09 vs. 0.05 h^−1^), S fraction (20.5% more; 235 vs. 195 g kg^−1^ DM), and EDDM (30.8% more; 522 vs. 399 g kg^−1^ DM), but lower (*p* < 0.05) RUDM (18.8% less; 497 vs. 612 g kg^−1^ DM) relative to alfalfa-HBG (Table 2). Thus, DM rumen degradability of alfalfa was greater relative to binary mixtures with AC Success HBG.

### 3.3. The Rumen Degradation Kinetics of CP

At the Saskatoon site, HiGest was lower (*p* < 0.05; 25.8% less) in soluble CP (241 ± 18 vs. 325 ± 17 g kg^−1^ CP; 25.8% less), but greater in degradable (D) fraction (581 ± 19 vs. 498 ± 17 g kg^−1^ CP; 16.6% more) (Table 4), whereas HiGest did not differ (*p* > 0.05) from AC Grazeland in T_0_ (avg. 1.71 ± 0.55 h), U (avg. 177 ± 5.5 g kg^−1^ CP), K_d_ (avg. 0.16 ± 0.01 h^−1^), and EDCP (avg. 689 ± 9.2 g kg^−1^ CP). In alfalfa monoculture, as the stage of maturity progressed, the undegradable fraction U and T_0_ increased (*p* < 0.05); however, EDCP was not changed (*p* = 0.831). HiGest-HBG had lower (*p* < 0.05) degradable CP fraction (14.1% less; 226 ± 18 vs. 263 ± 16 g kg^−1^ CP) but had greater soluble fraction S (27% more; 378 ± 18 vs. 297 ± 15 g kg^−1^ CP) relative to Grazeland-HBG, which was contradicted with those in monoculture. The HiGest-HBG did not differ (*p* > 0.05) from AC Grazeland-HBG in T_0_ (avg. 1.90 ± 0.43 h), U (avg. 417 ± 15 g kg^−1^ CP), K_d_ (avg. 0.11 ± 0.01 h^−1^), and EDCP (avg. 667 ± 43 g kg^−1^ CP). In binary mixtures, forage at the maturity stage 1 was greater in T_0_ (3.1 vs. 1.41 h), S (368 vs. 344 g kg^−1^ CP), and EDCP (528 vs. 506 g kg^−1^ CP) but was lower in D (246 vs. 252 g kg^−1^ CP) and U (385 vs. 404 g kg^−1^ CP) relative to those cut at stage 2, whereas forage cut at stage 3 did not differ (*p* > 0.05) from those cut at stage 1 or stage 2 in all measured parameters. Alfalfa showed higher (*p* < 0.05) K_d_ (53.4% more; 0.16 vs. 0.11 h^−1^), degradable fraction (220% more; 539 vs. 244 g kg^−1^ CP), and EDCP (139.6% more; 689 vs. 497 g kg^−1^ CP) than alfalfa-HBG. The lag time (avg. 1.9 ± 0.43 h) was not affected (*p* > 0.05) among binary mixtures (AC Grazeland-HBG vs. HiGest-HBG).

Forage in situ rumen degradation characteristics of CP at the Lanigan site are presented in Table 5. HiGest exhibited lower (*p* < 0.05) undegradable CP fraction U (28.0% less, 237 ± 15 vs. 329 ± 10.9 g kg^−1^ CP) and therefore had a greater EDCP (13.6% more; 608 ± 17.5 vs. 535 ± 8.3 g kg^−1^ CP) relative to AC Grazeland. HiGest was not different (*p* > 0.05) from AC Grazeland in S (avg. 299 ± 21.9 g kg^−1^ CP), K_d_ (avg. 0.10 ± 0.01 h^−1^) and T_0_ (avg. 1.04 ± 0.31 h). In alfalfa monoculture, as the stage of maturity progressed, the degradable fraction D decreased, and the undegradable fraction U and T_0_ increased (*p* < 0.05); however, EDCP was not changed (*p* = 0.185; avg. 571 ± 12.2 g kg^−1^ CP). In binary mixtures, K_d_ was increased (*p* = 0.017) from 0.04 to 0.09 h^−1^, but S was decreased from 211 to 148.5 g kg^−1^ CP (by 42.1%; *p* = 0.026) due to plant maturity advancing. However, there were no differences (*p* > 0.05) observed for T_0_ (the lag time; 1.76 ± 0.46 h), S (200 ± 15.0 g kg^−1^ CP), D fraction (308 ± 22.7 g kg^−1^ CP), as well as for EDCP (431.5 ± 32.9 g kg^−1^ CP) due to maturity stages. Likewise, in binary mixtures, T_0_ (avg. 1.76 ± 0.046 h), K_d_ (avg. 0.07 ± 0.008 h^−1^), D (avg. 307 ± 22.7 g kg^−1^ CP), U (avg. 492 ± 28.5 g kg^−1^ CP), as well as EDCP (avg. 357 ± 16.0 g kg^−1^ CP) were not affected (*p* > 0.05) by binary mixture types (AC Grazeland-HBG vs. HiGest-HBG). Alfalfa monocultures had higher (*p* < 0.05) K_d_ (0.10 vs. 0.07 h^−1^; 42.4% more), S (300 vs. 199 g kg^−1^ CP; 50.8% more), and EDCP (571 vs. 357 g kg^−1^ CP; 59.9% more) than alfalfa-HBG mixtures.

HiGest at stage 3 had greater (*p* < 0.05) EDCP (24.5% more; 668.1 vs. 535 g kg^−1^ CP) relative to Grazeland at stage 2. HiGest-HBG at stage 3 and Grazeland-HBG at stage 2 were similar (*p* > 0.05) in all measured parameters of CP degradation kinetics, excluding K_d_, which was lower (*p* < 0.05) in HiGest-HBG at stage 3. Overall, EDCP of HiGest were 97.8% and 113.7% of AC Grazeland alfalfa for Saskatoon and Lanigan sites, respectively, whereas EDCP of HiGest-HBG were slightly greater: 118.1% and 108.2% of AC Grazeland alfalfa-HBG for Saskatoon and Lanigan sites, respectively.

### 3.4. Rumen Degradation Kinetics of NDF

In situ rumen degradation kinetics of NDF of forages at the Saskatoon site are reported in Table 6. The HiGest contained lower (*p* < 0.05) undegradable NDF fraction (17.5% less; 450 ± 15 vs. 529 ± 16 g kg^−1^ DM) and hence greater D (16.8% more; 550 ± 15 vs. 471 ± 16 g kg^−1^ DM) as well as EDNDF (19.3% more; 340 ± 12 vs. 285 ± 13 g kg^−1^ DM) relative to those in AC Grazeland. The latter results suggest that HiGest may be a better alternative as a forage in terms of NDF degradability. In monoculture, as the maturity advanced, EDNDF decreased (by 18.4%) from 335 to 283 g kg^−1^ NDF for stage 1 and stage 3, respectively. In binary mixtures, HiGest-HBG tended to have greater NDF concentration (~5.2% greater; 260 ± 6.5 vs. 247 ± 6.5 g kg^−1^ DM; *p* = 0.105) or greater EDNDF (6.5% more; 163 ± 5.3 vs. 153 ± 4.5 g kg^−1^ DM; *p* = 0.037) relative to AC Grazeland-HBG. The HiGest-HBG was numerically higher in T_0_ (1.1 vs. 0.40 h) and K_d_ (0.04 vs. 0.03 h^−1^) (*p* > 0.05) relative to AC Grazeland-HBG. In binary mixtures, with maturity advancement, forage EDNDF concentration also declined (*p* < 0.05) and was 272, 257, and 231 g kg^−1^ NDF for stages 1, 2, and 3, respectively. Thus, the current study indicated that in both monoculture and binary mixtures, as the plant maturity advanced (stagers 1 to 3), EDDM, EDCP, and EDNDF declined.

In situ rumen degradation kinetics of NDF of forages at the Lanigan site are presented in Table 7. There was lower (*p* = 0.019) undegradable NDF fraction U (470 ± 17 vs. 517 ± 13 g kg^−1^ DM; 10% lower) and hence higher D (530 ± 17.9 vs. 483 ± 13 g kg^−1^ DM; 9.7% greater) and EDNDF (265 vs. 249 g kg^−1^ NDF, 6.5% greater) in HiGest relative to AC Grazeland. In alfalfa monoculture, as the stage of maturity progressed, the degradable fraction D decreased (*p* = 0.018) (by 6.3%) from 537 to 505.3 g kg^−1^ NDF for stage 1 and stage 3, respectively. In binary mixtures, as the stage of maturity advanced, EDNDF also declined (*p* < 0.001; either expressed as g kg^−1^ NDF or g kg^−1^ DM). Alfalfa had higher (*p* < 0.05) T_0_ (43% greater; 2.36 vs. 1.65 h), K_d_ (68.5% greater; 0.05 vs. 0.03 h^−1^), and U (52.2% greater; 493 vs. 324 g kg^−1^ NDF), but had lower NDF degradable fraction (33.4% lower; 506.9 vs. 676.4 g kg^−1^ NDF) relative to alfalfa-HBG (Table 7). HiGest at stage 3 was comparable with Grazeland at stage 2 in all measured parameters of NDF degradation kinetics, while HiGest-HBG at stage 3 and Grazeland-HBG at stage 2 were similar (*p* > 0.05) in EDNDF (either expressed as g kg^−1^ NDF or g kg^−1^ DM; data not shown), which indicated that HiGest maintains quality for a longer time. Thus, rumen degradation kinetics of alfalfa and alfalfa-HBG displayed some differences in both dark brown and black soil zones. Summarizing all four forages and three maturity stages, each percentage unit increase in lignin concentration decreased forage in situ effective degradability of NDF by 1.2 percentage units (data not shown).

## 4. Discussion

The study being described is the first of its kind to compare the rumen degradation kinetics of low-lignin HiGest and standard alfalfa cultivars under different cutting schedules in monocultures and binary mixtures in western Canada. 

The stage of maturity at cutting had a significant impact on the rumen degradation of both HiGest and Grazeland alfalfa. HiGest and Grazeland alfalfa did not differ (*p* > 0.05) in either NDF or CP concentrations in both sites. In general, HiGest had a greater EDDM and degradable DM fraction than AC Grazeland.

Overall, it appears that the HiGest alfalfa may have shown improved DM and NDF (neutral detergent fiber) degradability compared to the check alfalfa-AC Grazeland cultivar. The cultivar description by Alforex (2021) states that the Hi-Gest^®^360 alfalfa has a lower whole-plant lignin content (by 7–10%) compared to a non-selected elite commercial cultivar. This information is supported by the results of the current study. However, samples studied at both sites were from only 1 year, necessitating more research of samples from several years. In a similar fashion with the current study, our companion study [9] also revealed that Hi-Gest^®^ 360 alfalfa nutrient value was relatively greater in in vitro NDFD compared to conventional alfalfa cultivars. 

At the Saskatoon site, HiGest was numerically lower in acid detergent lignin (ADL; 10.2% lower; 59 vs. 65 g kg^−1^ DM; *p* = 0.57) relative to Grazeland. For monocultures, forages harvested at maturity stage 3 had greater ADL (36.4% more; 75 vs. 55 g kg^−1^ DM;) than those at maturity stage 1. Alfalfa monocultures had lower (*p* < 0.05) ADL than binary mixtures (Grazeland-HBG and HiGest-HBG). It appears that in both HiGest monoculture and HiGest-HBG binary mixtures, ADL concentrations were relatively lower and consistent up to stage 2 but increased rapidly thereafter. On the other hand, for Grazeland monoculture or Grazeland-HBG binary mixtures, ADL concentration gradually increased as plant maturity advanced. Additionally, averaged across two sites, HiGest alfalfa had 8.6% less ADL (65.1 vs. 59.5 g kg^−1^ DM) compared to Grazeland alfalfa [9].

The results of the current study are in agreement with others [17,18,19], where lignification has been reported to be the major factor limiting both the in vitro digestibility of plant cell wall polysaccharides and dry matter of whole-plant forage. However, the EDNDF of either Hi-Gest^®^360 or AC Grazeland alfalfa in the present study was lower than that reported by Yu et al. [2], who found 654 g kg^−1^ NDF in Pioneer and 617 g kg^−1^ NDF in Beaver alfalfa harvested at early bloom in the brown soil zone of Saskatchewan. This is probably because the quality of forage can be affected by various factors such as the cultivar [20] of the plant, the type of soil it is grown in, the climatic conditions it is exposed to [21], and the time of harvest or maturity stage [2,22]. As plants mature, leaf proportions decrease, stem proportions increase, stem cell wall concentrations increase, and whole plant nutritive value decreases. Among the factors listed, maturity at harvest can be easily altered and is critically important because it affects both quality and yield [23]. A study conducted by Hall et al. [24] investigated whether selecting for greater nutritive value resulted in delayed morphological development of alfalfa. The study concluded that there was no inadvertent selection for delayed morphological development when selection was made for greater nutritive value. A strong influence on forage digestibility and small decreases in the lignin concentration of forages can be expected to improve the fiber digestibility at any plant maturity stage [25]. This decline in degradability as maturity stage advanced in forage was evident in the current study. In addition, in the present study, there were very few differences on rumen degradation parameters among binary mixtures, which could be due to a number of reasons, as we speculated, including possibly lower presence or contribution of alfalfa in binary mixtures. In the current study, however, we were unable to determine botanical composition of the stand.

The key difference between grasses and legumes in terms of digestibility is that while tissues in legumes with thick lignified walls can be only marginally digested by rumen microbes, similarly thick lignified walls of grass tissues are extensively degraded, albeit slowly [26,27]. Concurring with the above, in the current study, EDNDF were greater in binary mixtures relative to monocultures of alfalfa at both sites. Moreover, in agreement with the current study, our previous work [11] found that smooth bromegrass (*Bromus inermis* Leyss.) alfalfa sun-cured hay (70:30 grass:alfalfa mixture) had 0.06 h^−1^ K_d_, 396 g kg^−1^ DM degradable, 235 g kg^−1^ DM soluble fraction, 369 g kg^−1^ undegradable fraction, and 463 g kg^−1^ EDDM.

## 5. Conclusions

The HiGest possessed an average of 13% greater EDNDF (267 vs. 302 g kg^−1^ NDF) relative to AC Grazeland. Pooled across two sites, HiGest alfalfa had greater EDDM concentration (avg. 571 vs. 544 g kg^−1^ DM; 5% more) relative to AC Grazeland alfalfa. The latter finding suggests that delaying the harvest of HiGest alfalfa by up to two weeks may not affect the degradable nutrients present in the crop. However, this statement is based on a single finding and may not be applicable to all growing conditions. Further research would be needed to confirm and generalize these results.

## Figures and Tables

**Table 1:** The CP and NDF (g kg^−1^ DM) of low-lignin alfalfa cultivar ‘Hi-Gest^®^360’ in monoculture and in binary mixtures with AC Success hybrid bromegrass (HBG) in Saskatoon and Lanigan, Saskatchewan.

			Site		
		Saskatoon		Lanigan	
Forage	Stage ^1^	CP	NDF	CP	NDF
Monoculture					
Grazeland	Stage 1	232	340	235	395
	Stage 2	196	309	210	440
	Stage 3	226	334	194	463
HiGest	Stage 1	216	360	228	411
	Stage 2	198	307	221	403
	Stage 3	236	329	207	451
SEM		19.0	16.0	6.9	17.0
Binary mixtures					
Grazeland-HBG	Stage 1	155	631	130	663
	Stage 2	121	607	95	664
	Stage 3	118	615	108	635
HiGest-HBG	Stage 1	133	64.3	126	666
	Stage 2	147	620	125	664
	Stage 3	123	612	121	629
SEM		14.1	9.9	12.4	10.4
Treatment contrasts					
Monoculture vs. Binary		<0.001	<0.001	<0.001	<0.001
Grazeland vs. HiGest		0.940	0.751	0.309	0.423
Grazeland-HBG vs. HiGest-HBG		0.802	0.341	0.207	0.848
Monoculture					
Stage 1 vs. Stage 2		0.679	0.259	<0.001	0.006
Stage 1 vs. Stage 3		0.111	0.019	0.035	0.283
Stage 2 vs. Stage 3		0.051	0.166	0.048	0.055
Binary mixtures					
Stage 1 vs. Stage 2		0.105	0.019	0.286	0.002
Stage 1 vs. Stage 3		0.466	0.019	0.170	0.925
Stage 2 vs. Stage 3		0.345	0.996	0.742	0.002

Note. ^1^ Forage cut at 3 maturity stages of alfalfa: stage 1 = alfalfa 10% bloom; stage 2 = 40% bloom; stage 3 = 100% bloom. Cutting dates were 8, 12, 19 July and 27 June, 8 July, and 29 July 2019 for the Saskatoon and Lanigan sites, respectively. CP, crude protein; NDF, neutral detergent fiber.

**Table 2:** Effects of cultivar, maturity, and their interaction on in situ rumen degradation kinetics of DM of alfalfa cultivars in monoculture and in binary mixtures with AC Success hybrid bromegrass (HBG) in Saskatoon, Saskatchewan Canada.

Forage	Stage *	T_0_ (h)	S (g kg^−1^ DM)	D (g kg^−1^ DM)	U (g kg^−1^ DM)	K_d_ (h^−1^)	EDDM (g kg^−1^ DM)
Monoculture							
Grazeland	Stage 1	1.06	267.8	447.8	282.9	0.14	599.6
	Stage 2	0.17	274.0	478.9	247.2	0.13	619.3
	Stage 3	0.46	264.8	430.1	305.1	0.16	590.0
HiGest	Stage 1	0.58	264.1	494.1	241.9	0.12	613.4
	Stage 2	0.57	286.4	488.3	225.3	0.16	647.2
	Stage 3	0.32	208.0	520.2	271.9	0.16	604.3
SEM		0.28	15.99	21.19	12.22	0.017	15.99
Binary mixtures							
Grazeland-HBG	Stage 1	0.18	174.8	440.6	384.7	0.05	400.8
	Stage 2	0.30	201.5	411.1	387.4	0.05	415.2
	Stage 3	0.00	179.0	409.1	411.9	0.06	402.8
HiGest-HBG	Stage 1	0.19	166.8	445.5	387.7	0.05	398.3
	Stage 2	0.36	222.2	387.3	390.6	0.05	422.1
	Stage 3	0.00	200.8	393.0	406.2	0.06	406.5
SEM		0.18	6.55	12.11	9.53	0.005	6.98
Treatment contrasts				*p* value			
Monoculture vs. Binary		0.014	<0.001	<0.001	<0.001	<0.001	<0.001
Grazeland vs. HiGest		0.767	0.239	0.014	0.006	0.844	0.047
Grazeland-HBG vs. HiGest-HBG		0.877	0.023	0.205	0.986	0.575	0.622
Monoculture							
Stage 1 vs. Stage 2		0.150	0.084	0.872	0.050	0.143	0.568
Stage 1 vs. Stage 3		0.136	0.387	0.581	0.049	0.569	0.116
Stage 2 vs. Stage 3		0.953	0.015	0.695	0.001	0.351	0.040
Binary mixtures							
Stage 1 vs. Stage 2		0.318	0.004	0.001	0.030	0.270	0.456
Stage 1 vs. Stage 3		0.413	<0.001	0.001	0.771	0.951	0.012
Stage 2 vs. Stage 3		0.080	0.001	0.863	0.053	0.296	0.052

Note. * Forage cut at 3 maturity stages of alfalfa: stage 1 = alfalfa 10% bloom; stage 2 = 40% bloom; stage 3 = 100% bloom. Cutting dates were 8, 12, 19 July 2019. T_0_, lag time; S, soluble fraction; K_d_, rate of degradation; D, potentially degradable fraction; U, undegradable fraction; EDDM, effective degradability; RUDM, rumen undegradable DM. SEM, standard error of the means within systems.

**Table 3:** Effects of cultivar, maturity, and their interaction on in situ rumen degradation kinetics of DM of alfalfa cultivars in monoculture and in binary mixtures with AC Success hybrid bromegrass (HBG) in Lanigan, Saskatchewan Canada.

Forage	Stage *	T_0_ (h)	S (g kg^−1^ DM)	D (g kg^−1^ DM)	U (g kg^−1^ DM)	K_d_ (h^−1^)	EDDM (g kg^−1^ DM)
Monoculture							
Grazeland	Stage 1	0.00	230.7	418.7	350.7	0.10	510.9
	Stage 2	0.17	195.8	425.3	379.0	0.10	473.3
	Stage 3	0.60	231.1	372.9	395.9	0.09	470.6
HiGest	Stage 1	0.62	216.0	478.8	305.2	0.10	529.2
	Stage 2	0.80	239.8	420.1	340.0	0.10	515.8
	Stage 3	1.23	298.5	386.0	315.6	0.07	515.9
SEM		0.340	18.86	20.90	13.50	0.006	12.00
Binary mixtures							
Grazeland-HBG	Stage 1	1.15	168.1	514.0	317.9	0.04	406.8
	Stage 2	1.14	230.8	473.8	295.4	0.03	389.5
	Stage 3	0.00	179.9	335.8	484.2	0.06	366.0
HiGest-HBG	Stage 1	1.74	179.7	469.4	350.9	0.05	410.5
	Stage 2	0.85	226.4	503.6	270.0	0.02	384.7
	Stage 3	0.15	185.3	332.1	482.6	0.07	373.0
SEM		0.360	9.96	16.64	21.75	0.005	8.01
Treatment contrasts				*p* value			
Monoculture vs. Binary		0.351	0.001	0.290	0.373	<0.001	<0.001
Grazeland vs. HiGest		0.007	0.049	0.202	<0.001	0.070	0.002
Grazeland-HBG vs. HiGest-HBG		0.613	0.552	0.628	0.907	0.540	0.639
Monoculture							
Stage 1 vs. Stage 2		0.045	0.040	0.005	0.056	0.005	0.031
Stage 1 vs. Stage 3		0.292	0.768	0.230	0.033	0.584	0.030
Stage 2 vs. Stage 3		0.293	0.023	0.055	0.784	0.014	0.987
Binary mixtures							
Stage 1 vs. Stage 2		0.002	0.318	<0.001	<0.001	0.004	<0.001
Stage 1 vs. Stage 3		0.233	<0.001	0.848	0.027	<0.001	0.001
Stage 2 vs. Stage 3		0.022	<0.001	<0.001	<0.001	<0.001	0.003

Note. * Forage cut at 3 maturity stages of alfalfa: stage 1 = alfalfa 10% bloom; stage 2 = 40% bloom; stage 3 = 100% bloom. Cutting dates were 27 June, 8 July, and 29 July 2019. T_0_, lag time; S, soluble fraction; K_d_, rate of degradation; D, potentially degradable fraction; U, undegradable fraction; EDDM, effective degradability; RUDM, rumen undegradable DM. SEM, standard error of the means within systems.

**Table 4:** Effects of cultivar, maturity, and their interaction on in situ rumen degradation kinetics of CP of alfalfa cultivars in monoculture and in binary mixtures with AC Success hybrid bromegrass (HBG) in Saskatoon, Saskatchewan Canada.

Forage	Stage *	T_0_ (h)	S (g kg^−1^ CP)	D (g kg^−1^ CP)	U (g kg^−1^ CP)	K_d_ (h^−1^)	EDCP (g kg^−1^ CP)	EDCP (g kg^−1^ DM)
Monoculture								
Grazeland	Stage 1	2.23	287.5	511.3	201.2	0.16	674.2	118.0
	Stage 2	1.41	303.8	542.3	153.9	0.15	706.5	106.0
	Stage 3	1.79	384.3	440.9	174.9	0.14	709.4	99.5
HiGest	Stage 1	2.11	280.8	527.5	191.6	0.16	681.8	114.5
	Stage 2	1.27	182.0	656.0	162.1	0.18	672.9	129.9
	Stage 3	1.46	260.3	558.3	181.4	0.18	690.3	131.9
SEM		0.378	22.60	20.20	11.97	0.031	23.90	12.24
Binary mixtures								
Grazeland-HBG	Stage 1	2.12	343.4	269.2	387.4	0.10	520.8	42.3
	Stage 2	1.72	285.1	291.4	423.5	0.09	467.9	35.1
	Stage 3	0.71	264.8	229.4	505.8	0.12	415.8	27.0
HiGest-HBG	Stage 1	4.06	394.4	222.1	383.4	0.11	534.5	29.1
	Stage 2	1.11	403.0	212.2	384.9	0.11	543.7	33.3
	Stage 3	1.69	337.6	242.9	419.4	0.11	500.3	29.9
SEM		1.032	27.90	29.06	33.01	0.025	27.60	6.24
Treatment contrasts				*p* value				
Monoculture vs. Binary		0.673	<0.012	<0.012	<0.001	<0.001	<0.001	<0.001
Grazeland vs. HiGest		0.505	<0.001	<0.001	0.860	0.320	0.689	0.095
Grazeland-HBG vs. HiGest-HBG		0.275	0.003	0.003	0.130	0.681	0.250	0.430
Monoculture								
Stage 1 vs. Stage 2		0.151	0.038	0.038	0.860	0.931	0.382	0.963
Stage 1 vs. Stage 3		0.036	0.026	0.026	<0.001	0.846	0.159	0.893
Stage 2 vs. Stage 3		0.445	<0.001	<0.001	0.100	0.914	0.031	0.857
Binary mixtures								
Stage 1 vs. Stage 2		0.038	0.028	0.028	0.030	0.654	0.037	0.257
Stage 1 vs. Stage 3		0.062	0.386	0.386	0.580	0.945	0.455	0.814
Stage 2 vs. Stage 3		0.800	0.145	0.145	0.100	0.594	0.149	0.363

Note. * Forage cut at 3 maturity stages of alfalfa: stage 1 = alfalfa 10% bloom; stage 2 = 40% bloom; stage 3 = 100% bloom. Cutting dates were 8, 12, 19 July 2019. T_0_, lag time; S, soluble fraction; K_d_, rate of degradation; D, potentially degradable fraction of CP; U, undegradable fraction of CP; EDCP, effective degradability of CP. SEM, standard error of the means within systems.

**Table 5:** Effects of cultivar, maturity, and their interaction on in situ rumen degradation kinetics of CP of alfalfa cultivars in monoculture and in binary mixtures with AC Success hybrid bromegrass (HBG) in Lanigan, Saskatchewan Canada in 2019.

Forage	Stage *	T_0_ (h)	S (g kg^−1^ CP)	D (g kg^−1^ CP)	U (g kg^−1^ CP)	K_d_ (h^−1^)	EDCP (g kg^−1^ CP)	EDCP (g kg^−1^ DM)
Monoculture								
Grazeland	Stage 1	0.00	213.7	465.0	321.3	0.09	516.0	100.4
	Stage 2	0.19	259.1	416.8	324.2	0.10	535.0	87.4
	Stage 3	1.70	384.3	271.5	244.3	0.08	554.2	65.1
HiGest	Stage 1	0.19	218.6	533.0	248.5	0.09	564.1	125.4
	Stage 2	0.82	272.9	460.5	266.6	0.11	592.1	102.1
	Stage 3	3.14	448.9	354.1	197.0	0.09	668.1	79.9
SEM		0.55	39.01	35.67	22.25	0.013	18.3	10.65
Binary mixtures								
Grazeland-HBG	Stage 1	1.13	247.5	287.4	465.1	0.05	388.2	38.1
	Stage 2	1.06	165.3	335.0	492.8	0.04	305.7	27.4
	Stage 3	1.29	164.6	258.2	577.2	0.11	331.2	25.7
HiGest-HBG	Stage 1	3.05	176.2	241.8	582.1	0.08	324.6	30.7
	Stage 2	1.16	286.1	440.1	273.9	0.04	465.1	53.8
	Stage 3	3.25	132.3	297.1	563.8	0.08	319.1	29.2
SEM		1.286	31.34	53.50	45.60	0.018	29.30	6.47
Treatment contrasts				*p* value				
Monoculture vs. Binary		0.200	0.007	<0.001	<0.001	0.004	<0.001	<0.001
Grazeland vs. HiGest		0.089	0.335	0.041	0.001	0.428	<0.001	0.054
Grazeland-HBG vs. HiGest-HBG		0.169	0.796	0.413	0.235	0.963	0.145	0.187
Monoculture								
Stage 1 vs. Stage 2		0.001	0.001	<0.001	0.001	0.718	0.002	0.002
Stage 1 vs. Stage 3		0.469	0.164	0.111	0.001	0.305	0.220	0.109
Stage 2 vs. Stage 3		0.002	0.002	0.003	0.235	0.173	0.020	0.054
Binary mixtures								
Stage 1 vs. Stage 2		0.870	0.031	0.785	0.233	0.121	0.176	0.300
Stage 1 vs. Stage 3		0.392	0.608	0.021	<0.001	0.109	0.208	0.375
Stage 2 vs. Stage 3		0.328	0.014	0.041	0.019	0.006	0.001	0.071

Note. * Forage cut at 3 maturity stages of alfalfa: stage 1 = alfalfa 10% bloom; stage 2 = 40% bloom; stage 3 = 100% bloom. Cutting dates were 27 June, 8 July, and 29 July 2019. T_0_, lag time; S, soluble fraction; K_d_, rate of degradation; D, potentially degradable fraction; U, undegradable fraction; EDCP, effective degradability of CP. SEM, standard error of the means within systems.

**Table 6:** Effects of cultivar, maturity, and their interaction on in situ rumen degradation kinetics of neutral detergent fiber (NDF) of alfalfa cultivars in monoculture and in binary mixtures with AC Success hybrid bromegrass (HBG) in Saskatoon, Saskatchewan Canada.

Forage	Stage *	T_0_	D (g kg^−1^ NDF)	U (g kg^−1^ NDF)	K_d_ (h^−1^)	EDNDF (g kg^−1^ NDF)	EDNDF (g kg^−1^ DM^)^
Monoculture							
Grazeland	Stage 1	2.06	488.6	511.4	0.09	303.7	102.7
	Stage 2	1.71	514.5	485.5	0.07	300.2	92.3
	Stage 3	0.55	410.3	589.7	0.08	252.4	83.4
HiGest	Stage 1	0.67	588.6	411.4	0.08	366.3	131.7
	Stage 2	1.76	562.4	437.6	0.08	341.5	105.1
	Stage 3	2.00	499.4	500.7	0.09	313.7	102.9
SEM		0.59	17.96	17.96	0.010	20.69	7.37
Binary mixtures							
Grazeland-HBG	Stage 1	0.55	623.6	376.4	0.04	263.2	166.2
	Stage 2	0.56	603.5	396.5	0.04	254.1	154.4
	Stage 3	0.07	599.0	401.0	0.03	225.8	138.9
HiGest-HBG	Stage 1	1.49	607.9	392.2	0.04	280.5	180.4
	Stage 2	1.42	582.2	417.8	0.04	260.0	160.8
	Stage 3	0.29	582.2	417.8	0.04	236.2	144.6
SEM		0.477	17.25	17.26	0.003	8.72	5.73
Treatment contrasts				*p* value			
Monoculture vs. Binary		0.029	<0.001	<0.001	<0.001	<0.001	<0.001
Grazeland vs. HiGest		0.941	<0.001	<0.001	0.675	0.005	0.004
Grazeland-HBG vs. HiGest-HBG		0.098	0.222	0.222	0.053	0.105	0.037
Monoculture							
Stage 1 vs. Stage 2		0.884	<0.001	<0.001	0.942	0.024	0.005
Stage 1 vs. Stage 3		0.541	0.993	0.993	0.317	0.506	0.024
Stage 2 vs. Stage 3		0.451	0.001	0.001	0.352	0.087	0.466
Binary mixtures							
Stage 1 vs. Stage 2		0.093	0.164	0.164	0.014	<0.001	<0.001
Stage 1 vs. Stage 3		0.948	0.203	0.203	0.573	0.082	0.005
Stage 2 vs. Stage 3		0.105	0.896	0.896	0.044	0.003	0.004

Note. * Forage cut at 3 maturity stages of alfalfa: stage 1 = alfalfa 10% bloom; stage 2 = 40% bloom; stage 3 = 100% bloom. Cutting dates were 8, 12, and 19 July 2019. T_0_, lag time; S, soluble fraction; K_d_, rate of degradation; D, potentially degradable fraction of NDF; U, undegradable fraction of NDF; EDNDF, effective degradability of NDF. SEM, standard error of the means within systems.

**Table 7:** Effects of cultivar, maturity, and their interaction on in situ rumen degradation kinetics of neutral detergent fiber (NDF) of alfalfa cultivars in monoculture and in binary mixtures with AC Success hybrid bromegrass (HBG) in Lanigan, Saskatchewan Canada.

Forage	Stage *	T_0_ (h)	D (g kg^−1^ NDF)	U (g kg^−1^ NDF)	K_d_ (h^−1^)	EDNDF (g kg^−1^ NDF)	EDNDF (g kg^−1^ DM)
Monoculture							
Grazeland	Stage 1	1.26	491.9	508.1	0.06	262.7	103.9
	Stage 2	2.99	481.6	518.4	0.06	250.9	111.2
	Stage 3	1.69	476.7	523.3	0.05	234.1	108.9
HiGest	Stage 1	1.89	581.9	418.1	0.06	304.1	125.0
	Stage 2	3.63	475.7	524.3	0.06	262.3	105.8
	Stage 3	2.72	533.8	466.2	0.04	227.5	102.0
SEM		0.949	24.10	24.10	0.008	14.65	7.87
Binary mixtures							
Grazeland-HBG	Stage 1	2.67	721.6	278.4	0.04	312.6	207.4
	Stage 2	2.25	757.3	242.7	0.02	225.2	149.2
	Stage 3	0.64	517.9	482.1	0.04	218.6	138.7
HiGest-HBG	Stage 1	2.46	728.6	271.4	0.04	304.7	203.2
	Stage 2	1.92	812.8	187.2	0.02	215.8	143.2
	Stage 3	0.00	520.2	479.8	0.04	223.4	140.7
SEM		0.52	42.50	42.50	0.004	11.13	8.11
Treatment contrasts				*p* value			
Monoculture vs. Binary		0.144	<0.001	<0.001	<0.001	0.567	<0.001
Grazeland vs. HiGest		0.328	0.019	0.019	0.726	<0.043	<0.189
Grazeland-HBG vs. HiGest-HBG		0.342	0.543	0.543	0.770	0.650	0.665
Monoculture							
Stage 1 vs. Stage 2		0.507	0.169	0.168	0.071	0.002	0.258
Stage 1 vs. Stage 3		0.081	0.018	0.018	0.865	0.069	0.452
Stage 2 vs. Stage 3		0.251	0.243	0.243	0.097	0.078	0.692
Binary mixtures							
Stage 1 vs. Stage 2		<0.001	<0.001	<0.001	0.721	<0.001	<0.001
Stage 1 vs. Stage 3		0.344	0.179	0.178	0.001	<0.001	<0.001
Stage 2 vs. Stage 3		0.003	<0.001	<0.001	0.002	0.963	0.411

Note. * Forage cut at 3 maturity stages of alfalfa: stage 1 = alfalfa 10% bloom; stage 2 = 40% bloom; stage 3 = 100% bloom. Cutting dates were 27 June, 8 July, and 29 July 2019. T_0_, lag time; S, soluble fraction; K_d_, rate of degradation; D, potentially degradable fraction of NDF; U, undegradable fraction of NDF; EDNDF, effective degradability of NDF. SEM, standard error of the means within systems.

## Data Availability

Raw data are available upon request to H.A. Lardner.
